# Predicting the placement of biomolecular structures on AFM substrates based on electrostatic interactions

**DOI:** 10.3389/fmolb.2023.1264161

**Published:** 2023-11-28

**Authors:** Romain Amyot, Kaho Nakamoto, Noriyuki Kodera, Holger Flechsig

**Affiliations:** ^1^ JSPS International Research Fellow, Kanazawa, Ishikawa, Japan; ^2^ Nano Life Science Institute (WPI-NanoLSI), Kanazawa University, Kanazawa, Ishikawa, Japan

**Keywords:** AFM (atomic force microscope), sample placement, electrostatic interaction, protein dynamics analysis, image analysis, software application

## Abstract

Atomic force microscopy (AFM) and high-speed AFM allow direct observation of biomolecular structures and their functional dynamics. Based on scanning the molecular surface of a sample deposited on a supporting substrate by a probing tip, topographic images of its dynamic shape are obtained. Critical to successful AFM observations is a balance between immobilization of the sample while avoiding too strong perturbations of its functional conformational dynamics. Since the sample placement on the supporting substrate cannot be directly controlled in experiments, the relative orientation is *a priori* unknown, and, due to limitations in the spatial resolution of images, difficult to infer from *a posteriori* analysis, thus hampering the interpretation of measurements. We present a method to predict the macromolecular placement of samples based on electrostatic interactions with the AFM substrate and demonstrate applications to HS-AFM observations of the Cas9 endonuclease, an aptamer-protein complex, the Monalysin protein, and the ClpB molecular chaperone. The model also allows predictions of imaging stability taking into account buffer conditions. We implemented the developed method within the freely available BioAFMviewer software package. Predictions based on available structural data can therefore be made even prior to an actual experiment, and the method can be applied for post-experimental analysis of AFM imaging data.

## Introduction

High-speed atomic force microscopy (HS-AFM) allows direct observation of biomolecules during their operation under near-physiological conditions (Ando et al., 2013; Ando et al., 2014), with its applications having significantly advanced the understanding of biological processes at the nanoscale (Ando, 2022). Furthermore, by the combination of HS-AFM and computational modeling even atomistic details of protein function can be inferred (Flechsig and Ando, 2023).

An AFM experiment requires the biological sample to be first deposited on a supporting surface, after which scanning of the molecular surface by a probing tip proceeds to record a topographic image of its shape at a spatial resolution of ∼1–2 nm in the lateral direction and typically less than 0.5 nm in the vertical direction. It is important to understand that the observation of single proteins under HS-AFM is a delicate balance between immobilizing the structure on the supporting surface while at the same time preventing too strong perturbations by immobilization. i.e., stable and steady scanning of the protein by the probing tip requires sufficient fixation on the surface through molecular interactions. However, the reliable observation of protein activity rests on the assumption that such interactions (which are not present under physiological conditions or *in vitro* experiments) do not significantly interfere with the functional conformational dynamics of the protein.

The process of placing a biomolecular sample on the supporting surface and controlling its proper attachment is a challenge at the very start of every HS-AFM observation. Mica, silicon and highly oriented pyrolytic graphite (HOPG) are often used as the supporting substrates. Because of its surface flatness at the atomic level over a large area and easy to prepare surface modifications, the negatively charged mica substrate is most frequently used. It is possible to modify mica with specific molecules [e.g., 3-Aminopropyltriethoxysilane (APTES), poly-L-lysin (PLL) or lipid-bilayers], hence altering the charge properties. Furthermore, by the chemical composition of the buffer interactions between the sample and substrate can be modified. Such surface modifications are often critical for successful AFM observations of protein structures and their functional motions (Shlyakhtenko et al., 2010; Yamamoto et al., 2010; Endo, 2019).

It would clearly be valuable to have methods available that can predict the placement of biomolecular structures on the supporting substrate even prior to an AFM experiment being performed, and, on the other side, to facilitate the post-experimental analysis of recorded images to better understand measured AFM topographies. We report here the development of a computational framework to address such issues based on an electrostatic interaction model and demonstrate various applications. The method is implemented within the freely available BioAFMviewer software package (Amyot and Flechsig, 2020; Amyot et al., 2023).

## Materials and methods

### Electrostatic interaction model

Electrostatic interactions between the sample and the substrate can be described by the Debye-Hückel potential

$$V_{ele}=\sum_{i<j}\frac{q_{i}q_{j}}{4\pi \epsilon_{0}\epsilon_{k}d_{ij}}e^{-d_{ij}/\lambda_{D}},\lambda_{D}=\sqrt{\frac{\epsilon_{0}\epsilon_{k}k_{B}T}{2N_{A}e^{2}I}}$$ (1)

which represents Coulomb interactions of point charges *q*_*i*_ and *q*_*j*_ separated by the spatial distance *d*_*ij*_, effectively screened over the Debye length $\lambda_{D}$ . The Debye length can vary between 2.1 nm for ionic strength *I* = 20 mM and 0.8 nm for *I* = 150 mM (ionic strength under physiological conditions).

### Substrate modelling

We considered three different AFM substrates, i.e., the mica surface, APTES-mica, and lipid bilayer surfaces and for the purpose of this study construct simplified 2D models of them. We employ an atomic model of the cleaved surface of the muscovite mica crystal structure (Fukuma et al., 2010). The oxygen atoms have charges q=−2e and although silicon atoms have charges +4e , we assign them effective charges q=+2e to roughly account for the charge compensation by the fourth oxygen atom which is covalently bound to each silicon atom but is not part of the cleaved surface. The charge density of this model reproduces the experimentally known value for mica −e/0.48 nm^2^ (Uchihashi et al., 2018a). Based on the known charge density we also considered a coarse-grained mica model which has point-like charges q=−e placed along a regular lattice with spacing $\sqrt{0.48}$ nm.

To construct a minimal 2D model of the APTES-mica surface, we first computed the average gyration radius of ten different 3D APTES conformers available from the PubChem website as $R_{g}=3.4$ Å. Viewing individual APTES molecules as spheres, it would be possible to construct a regular lattice with a spacing of ${2R}_{g}$ . However, since such an arrangement would result in an unrealistic charge distribution under commonly used APTES concentrations (i.e., it would practically represent the mica lattice geometry with positive charges), we instead imposed a regular grid of point-like positive charges with a spacing of $R_{g}$ , assuming contributions from internal conformational changes during the assembly process which are unknown. The charge density of this model is larger by factor of about 4 compared to the mica model.

To model lipid-bilayers self-assembled on the mica surface, we impose the 2D hexagonal packed geometry of lipid headgroups inferred from previous high-resolution AFM imaging (Higgins et al., 2006) with a measured intermolecular spacing of 0.51 nm, and view each group as a point-like particle with either neutral, positive, or negative charge, reflecting the most commonly used lipid types (DPPC, DPPE, DOPC lipids q=0 ; DPTAP type q=+e ; DOPS type q=−e ). The fraction of charges can be specified and a random distribution along the lattice will be assumed. The *Electrostatics Application* within the BioAFMviewer allows to consider customized models of AFM substrates with specified lattice geometry parameters and adjusted ratio of charged particles too. Hence, a library of substrate surface models becomes available.

### Electrostatic potential calculation

To construct the 3D surface of a macromolecular structure, the well-known *marching cubes* discretization algorithm (Lorensen and Cline, 1987) was employed, representing it by a set of triangles used for graphical rendering. For each triangle the electrostatic potential is evaluated at the center of mass (vertex) as the sum of Coulomb potentials arising from partial charges of all atoms. Hence, the potential for vertex i is $V_{i}={\left(4\pi \epsilon_{0}\right)}^{-1}\sum_{j}q_{j}/d_{ij}$ , where $q_{j}$ is the partial charge of atom j , $d_{ij}$ is the distance between vertex i and atom j , and $\epsilon_{0}$ is the vacuum permittivity. This allows to compute the surface electrostatic potential for a given PDB structure. The *Electrostatics Application* within the BioAFMviewer implements a graphical representation of the generated 3D molecular surface where values of the electrostatic potential are visualized via a color scale. In the applications for Cas9, aptamer-CYP24, Monalysin, and ClpB, the partial charges of atoms were computed at pH value 7.0 condition.

### Orientation sampling, energy landscape and prediction

For sampling 3D rigid-body orientations of a biomolecular structure, we discretized the search space evenly using the Fibonacci lattice algorithm (Swinbank and Purser, 2006; Gonzalez, 2010). In the applications for Cas9, aptamer-CYP24, Monalysin, and ClpB, sampling was performed for a set of 2000 conformations. The *Electrostatics Application* within the BioAFMviewer gives a choice for this number. For each single orientation, direct contact to the AFM substrate was always assumed, and the electrostatic interaction energy between all substrate point charges and the biomolecular surface was computed according to the Debye-Hückel form (Eq. 1). For a single substrate point charge *q*_*i*_ the electrostatic potential energy is $E_{i}=\frac{1}{\epsilon_{k}}\frac{1}{A_{total}}\sum_{j}A_{j}V_{j}q_{i}e^{-d_{ij}/\lambda_{D}}$ , where *V*_*j*_ is the electrostatic potential value of triangle *j* (with area *A*_*j*_) on the sample surface, *A*_*total*_ is the total sample surface area, *d*_*ij*_ is the distance between the triangle center of mass and the charge *q*_*i*_, and $\epsilon_{k}$ is the dimensionless relative permittivity. Area weights were introduced because the *marching cubes* algorithm does not discretize the sample surface evenly, resulting in a heterogeneous density of vertices (triangle center of mass). To remove the density dependence in the electrostatic potential energy, individual contributions of triangles were therefore weighted considering their fraction to the total surface area. After completed sampling, a visualization of the energy landscape in the space of latitude and longitude angles ∅,θ (characterizing the sample orientation relative to the substrate) was obtained.

The interpretation of pathways in this landscape is as follows. For any fixed value of the angle θ , changes in the angle ∅ always correspond to rotation of the biomolecular sample around the *y*-axis of the AFM substrate plane. Considering any fixed value of ∅ , changes in the angle θ correspond to the sample rotating around an axis which is given by the AFM substrate *x*-axis co-rotated by the ∅ value. We note that for the sake of better interpretation we always plot the energy landscape in the space of both latitude and longitude angles ∅,θ covering the 360° range. For presentation purposes, we shift the energy scale such that the global minimum has zero energy. Furthermore, since the gross approximations applied in our modelling do not allow to infer a realistic magnitude of electrostatic interaction energies between the sample and the AFM substrate (see Discussion), we use rescaled dimensionless values such that the maximum value equals unity. For relative comparison in the case of the toy sphere models and in the ClpB case (high salt versus low salt buffer conditions) a common rescaling was used. The prediction of most favorable placements on the substrate was based on identifying the minima of the landscape. In the software the top five candidates, corresponding to the five lowest values, are displayed.

### BioAFMviewer workflow

The developed methods are implemented via the *Electrostatics Application* tool within the BioAFMviewer interactive software interface. To use this application, the user has to upload a PQR file of the biomolecular structure which, as compared to a regular PDB file, contains information about partial charges of atoms. For a given biomolecular structure such data can be conveniently obtained using, for example, the PDB2PQR application (Jurrus et al., 2018) (server https://server.poissonboltzmann.org/pdb2pqr), which also considers calculations at variable pH value and for different force fields. After loading the PQR file, the biomolecular structure can be displayed in the surface representation with coloring according to the calculated electrostatic surface potential. In the *Electrostatics Application* tool, the user can either choose the AFM substrate from a list of commonly employed examples with preset charge distribution or provide alternative values. The prediction of electrostatically favorable biomolecular orientations can be started after fixing the size of the sampling set. After completed sampling, the landscape of electrostatic interaction energies is displayed in an interactive window, which allows to access by mouse-click any point and visualize the corresponding molecular placement on the AFM model substrate in the front view, bottom view, and scanning view perspectives, together with the simulated AFM image.

### Simulation atomic force microscopy

We have employed simulation AFM to compare the results from electrostatic predictions with measured HS-AFM topographies. Simulation AFM computationally emulates AFM scanning to convert available biomolecular structures into simulated AFM images that can be correlated with experimentally obtained images. It is based on the non-elastic collisions of a rigid cone-shaped tip with a rigid Van-der-Waals sphere atomistic model of the biomolecular structure. For details we refer to our previous work (Amyot and Flechsig, 2020). Simulation AFM calculations were performed within the BioAFMviewer software platform (Amyot and Flechsig, 2020; Amyot et al., 2023).

### HS-AFM imaging of substrates

A mica substrate (∼0.1 mm in height and 1.5 mm in diameter) was glued with epoxy on the top of a cylindrical glass stage (2 mm in height and 2 mm in diameter). The mica surface was prepared by cleaving the top layers of mica disk, which was then immediately imaged with HS-AFM in the observation buffer (10 mM Tris-HCl, pH 7.5). The 3-aminopropyltriethoxysilane modified mica (APTES-mica) surface was prepared as previously reported (Yoshimi et al., 2022), using an APTES solution diluted to 0.1% with MilliQ-water, after which the surface was imaged with HS-AFM in the observation buffer.

HS-AFM imaging was performed in the tapping mode using small cantilevers (BLAC10DS-A2, Olympus) (resonant frequency, ∼0.5 MHz in water; quality factor, ∼1.5 in water; spring constant, ∼0.1 N·m^−1^). The cantilever’s free oscillation amplitude *A*_0_ and set-point amplitude were set at ∼2 nm and ∼0.9 × *A*_0_, respectively. The scan size and pixel size for each AFM image are 80 × 80 nm^2^ and 160 × 160 pixel, respectively. The frame rate for the mica and APTES-mica surfaces were 0.5 and 0.25 s/frame, respectively.

For image analysis, a low-pass filter to remove spike noise and a flattening filter to make the xy-plane flat were applied to each image. The height distributions of the mica and APTES-mica surfaces were fitted with single and double Gaussian functions, respectively. For the mica, the center of the Gaussian peak was set to 0 nm. For the APTES-mica, the center of the first Gaussian peak, which corresponds to the mean height of the mica surface, was set to 0 nm.

## Results

**FIGURE 1:**
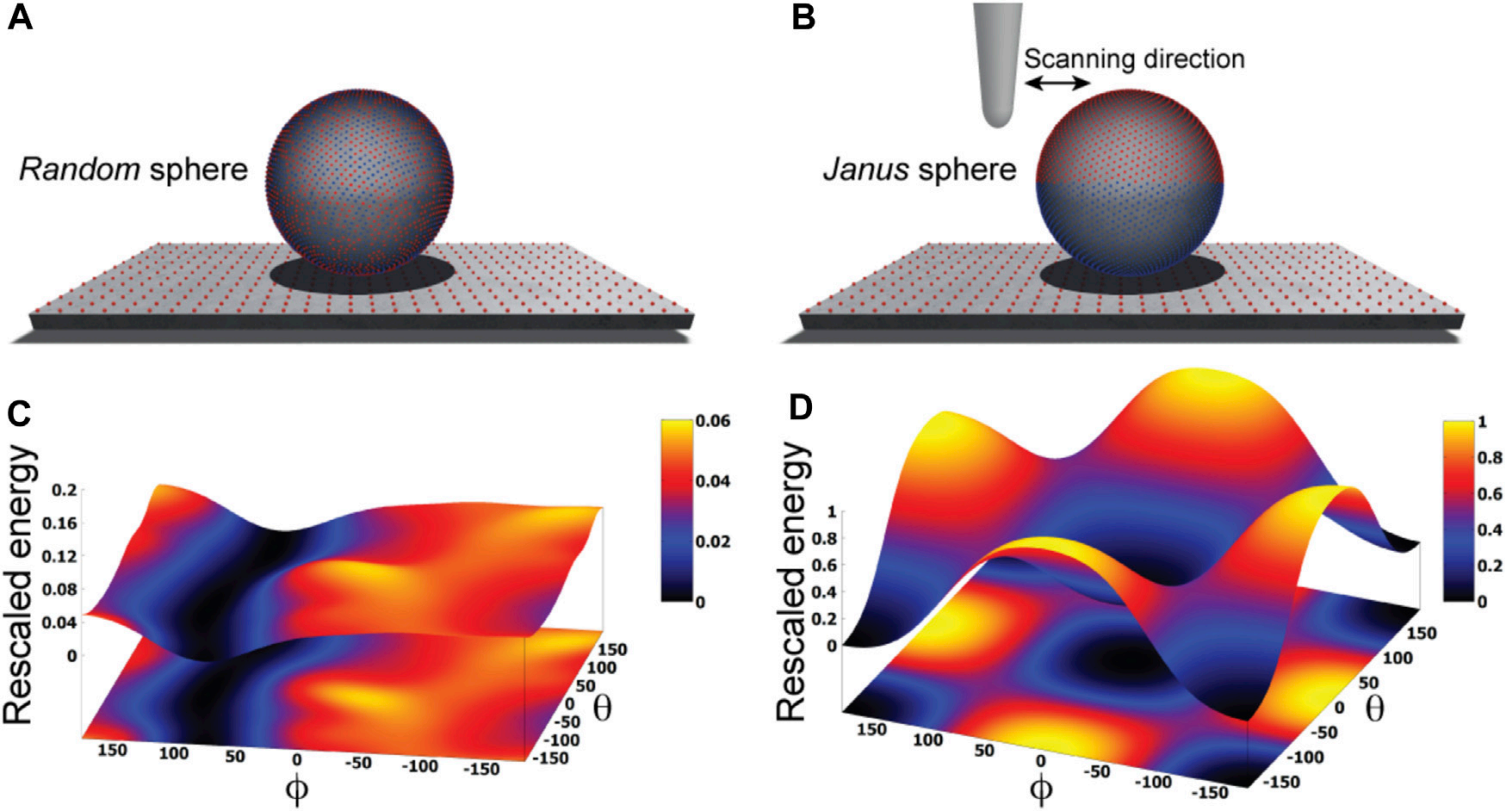
Idealized toy samples. Sphere with 5000 surface point charges of randomly picked sign **(A)** versus a *Janus* sphere carrying opposite charges separated on either hemisphere **(B)**, each placed on a 2D substrate plate which has point charges arranged along a regular grid. Blue and red colors represent positive and negative unit charges, respectively. The landscape of electrostatic interaction energies for the *random* sphere **(C)** and *Janus* sphere **(D)**, respectively. In both plots a common energy scale was used by rescaling.

We illustrate our approach by first considering an idealized situation in which the sample is viewed as a perfectly spherical solid object. Two cases are distinguished. In one case point-like unit charges q_i_ with randomly picked sign are distributed uniformly on the surface of the sphere, while in the second case the two hemispheres carry opposite charges creating a *Janus sphere* (Figures 1A, B). The AFM supporting substrate is modelled as a 2D solid plate which has point-like charges placed along a regular grid.

Here, we employ a simplified description resting on the approximation that the sample is placed on top of the AFM substrate (Figure 1) and its atomistic structure does not undergo any internal conformational changes. We then systematically explore molecular orientations of the sample relative to the substrate by performing rigid-body rotations in 3D space, recording the electrostatic interaction energy for each instantaneous configuration (see Methods). Thus, a landscape of electrostatic interaction energies in the space of appropriately chosen coordinates can be constructed, which shall allow an interpretation of the stability of sample-substrate interactions. We employed the latitude and longitude angles ∅,θ to characterize the sample orientation in 3D space (see Methods). A landscape with multiple minima separated by shallow barriers would indicate rather unstable placement of the sample on the surface. This situation is demonstrated for the case of the randomly charged sphere (Figure 1C) and its interpretation is that a plethora of possible molecular orientations with respect to the stage are practically as likely while a single stable configuration cannot be formed. The situation is very much different for the Janus sphere, where the landscape shows a highly symmetric shape of a funnel leading into a deep valley with a global minimum that corresponds to a single most stable configuration (Figure 1D). In this arrangement, the positive charged hemisphere is aligned towards the negatively charged mica substrate contacting it around the pole, and the negatively charged hemisphere is pointed upwards. Deviations from this stable state correspond to uphill motions in the energy landscape, which require forces $F_{\phi }=-\partial V/\partial \phi$ and $F_{\theta }=-\partial V/\partial \theta$ . Since changes in the angle ϕ correspond to a rotation of the sample around an axis within the supporting substrate, the component $F_{\phi }$ has an intuitive meaning of the force that would be caused by the AFM tip in the horizontal scanning direction (see Figure 1B).

**FIGURE 2:**
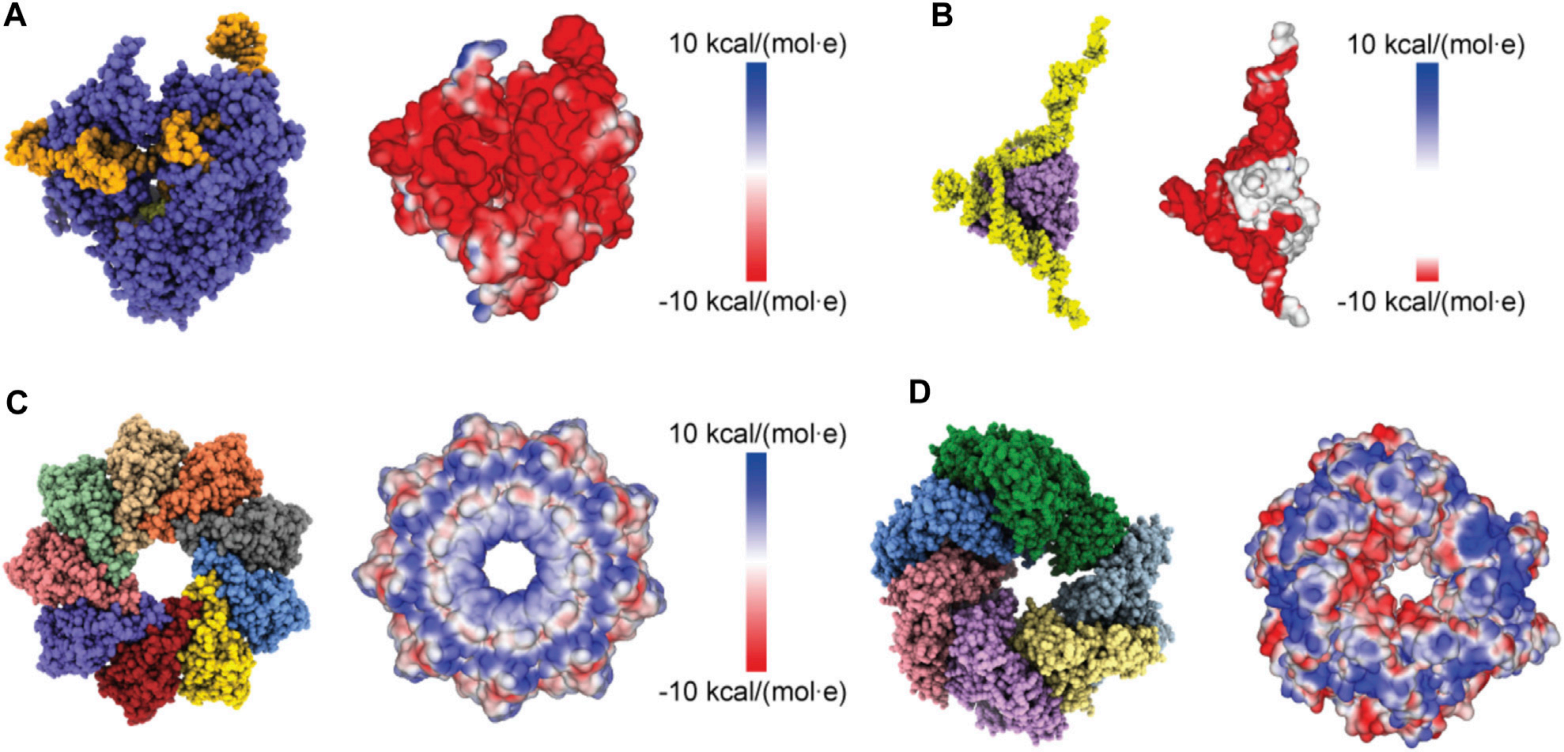
Protein surface electrostatic potential. **(A)** Molecular structure of the Cas9-RNA-DNA endonuclease complex (left, PDB 4OO8) and the computed surface representation with a coloration representing electrostatic potential values (right). **(B)** Molecular structure of the aptamer-Cyp24A protein complex (left) and the colored surface representation (right). **(C)** Molecular structure of the Monalysin protein (left, PDB 4MJT with removed N-terminal segments) and the colored surface (right). **(D)** Molecular structure of the Hsp104 hexamer [left, PDB 5KNE with reconstructed amino acid side chains (Krivov et al., 2009) and truncated N-terminal domains] and the colored surface representation (right).

Proceeding with applications to biomolecular structures, we have first applied a method to compute the electrostatic potential on the molecular surface based on the Coulomb contributions of all amino acids (see Methods). For a given structure, we thus obtained a graphical representation of its molecular surface where values of the electrostatic potential are mapped on a color scale (Figure 2). Similar graphical representations are typically provided by standard molecular viewers such as ChimeraX (Pettersen et al., 2021), Pymol (Lilkova, 2015) and others.

We have considered four different examples of proteins, a Cas9 endonuclease, an aptamer-protein complex, the Monalysin protein, and the molecular chaperone ClpB. For three cases we have previously applied simulation atomic force microscopy and automatized rigid-body fitting within the BioAFMviewer software package to predict the molecular orientation from resolution-limited HS-AFM topographies (Amyot and Flechsig, 2020; Amyot et al., 2022; Amyot et al., 2023), which allowed to disambiguate the arrangement of functional domains and to identify the relative orientation of domains with respect to bound nucleic acids.

Here, we now apply the electrostatic interaction model to predict the 3D molecular placement of the sample on an AFM substrate prior to an actual experiment being performed. We also discuss predictions for the stability of observations. To validate model predictions, we furthermore provide comparison to images from HS-AFM experiments.

### Modelling of AFM substrates

**FIGURE 3:**
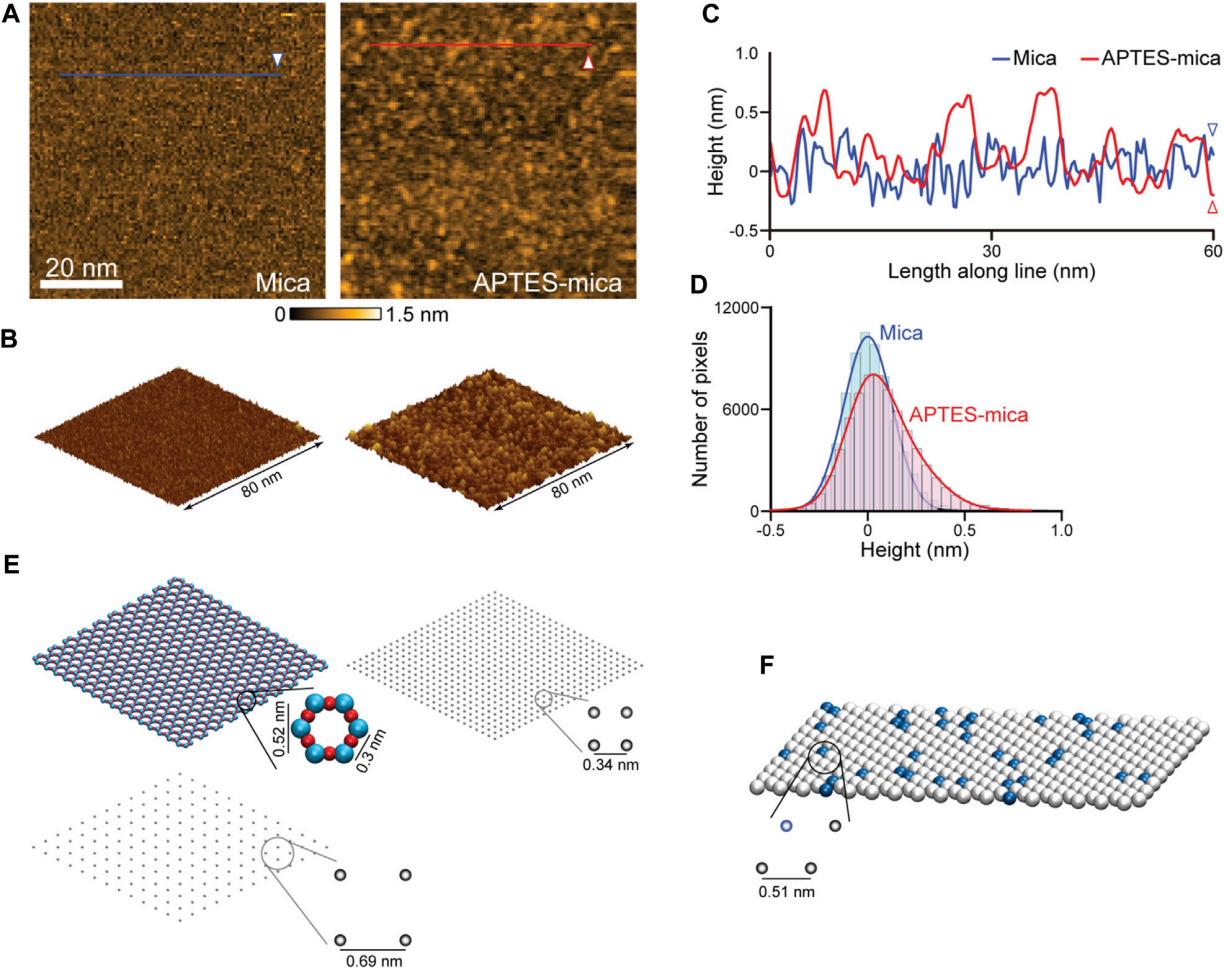
Models of AFM substrates. **(A)** HS-AFM images of the mica substrate (left) and APTES-mica (right). **(B)** Corresponding topographies in the 3D perspective view. **(C)** Height profiles measured along the two lines indicated in panel **(A)**. **(D)** Histograms of measured topography heights obtained from the mica and APTES-mica images. **(E)** Left: The atomic resolution model of the muscovite mica cleaved surface (top), and a coarse-grained regular lattice model of point-like negative charges (bottom). Right: The simplified APTES-mica regular lattice model of point-like negative charges. **(F)** Lipid-bilayer model with the hexagonal packing of lipid headgroups (illustrated as beads). In this example a 1:10 ratio of positive charged (blue color) and neutral charged lipids is illustrated.

Relevant for our study is the modelling of three different AFM substrates. Because of its surface flatness at the atomic level over a large area, the negatively charged mica substrate is most frequently used. The mica surface modified with APTES molecules is preferred when imaging, e.g., proteins complexed with nucleic acids, because in water the NH_2_ group of an APTES molecule is protonated to the positively charged NH_3_^+^ under typical pH conditions. The processes by which APTES molecules interact with mica and the formation of the coated surface are largely unexplored. We have performed HS-AFM imaging of the APTES-mica and compared it with an image of bare mica (see Figures 3A, B). Under typically used molecular concentration of APTES, the surface appears much more rugged showing irregular accumulations of larger blobs and indentations (Figures 3A, C). While the distribution of measured topography heights for bare mica is Gaussian, that obtained from the APTES-mica image is clearly asymmetric towards larger height values (Figure 3D). These observations indicate the complexity underlying the formation of the ATPES-mica substrate, possibly involving aggregation of individual APTES molecules and inhomogeneous binding to the Mica surface.

For the mica surface, we consider an atomic resolution model as well as a simplified lattice model. In the framework of our study, we can only formulate a simplified minimal model of the ATPES-mica surface viewing it as a regular lattice of point-like positive charges. The substrate models are illustrated in Figure 3E. For details we refer to the Methods section.

The third model substrate is that of a lipid bilayer self-assembled on the mica surface. We employ a model of the lipid headgroups in the hexagonally packed geometry inferred from previous high-resolution AFM imaging (Fukuma et al., 2010) (see Figure 3F). Since commonly a mixture of various lipid types is used in experiments, we consider a combination of neutral charges (e.g., DPPC, DPPE lipids) and positive charged headgroups (e.g., DPTAP lipid type), see Methods.

### Cas9-RNA-DNA complex

**FIGURE 4:**
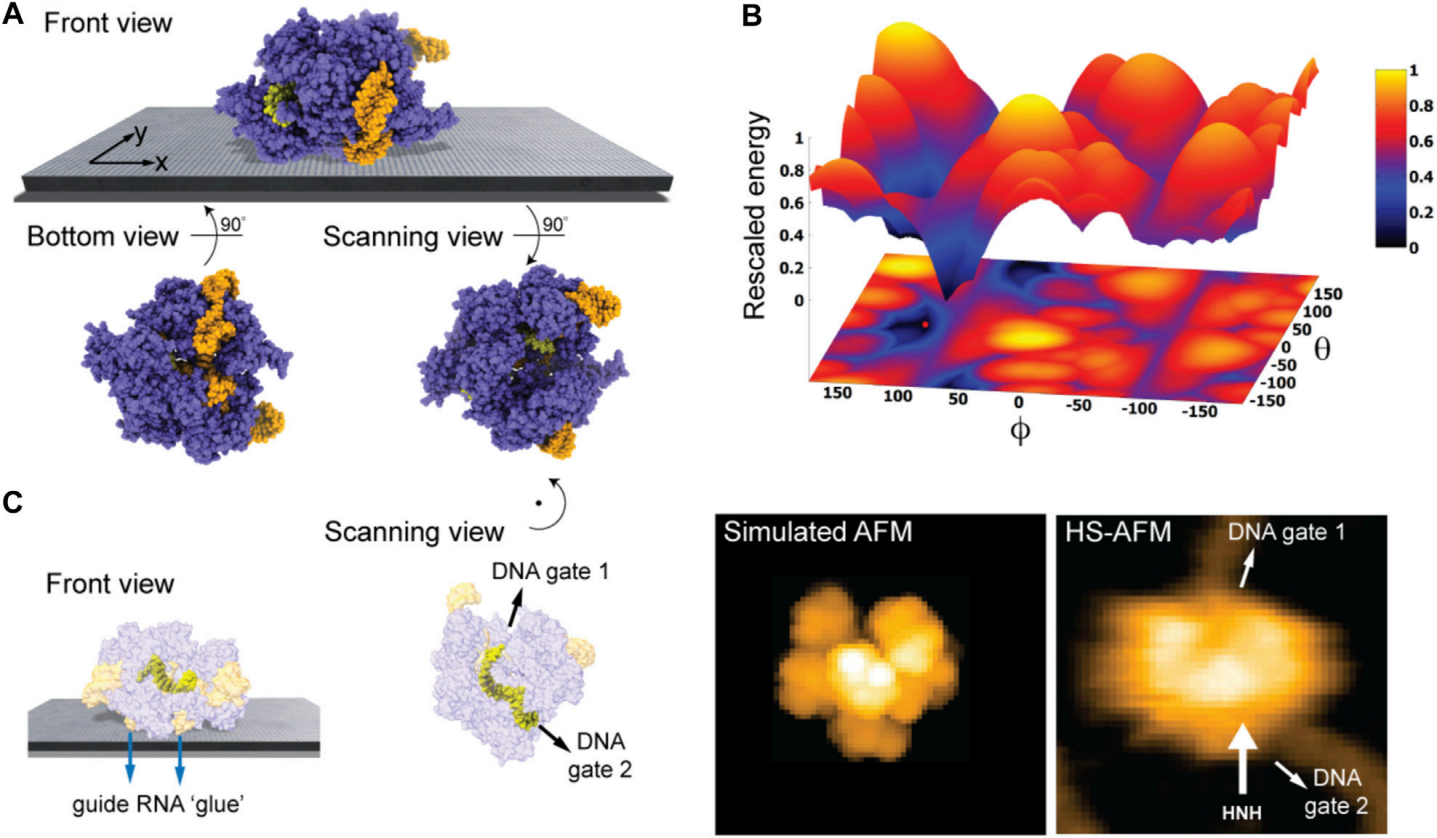
Cas9-RNA-DNA complex. **(A)** Predicted orientation of the protein complex shown on the supporting surface in the front view. Additionally, the bottom view perspective displays the structure facing the surface and the scanning view shows the side probed by the AFM tip. The guide RNA strand and target DNA are colored in orange and yellow, respectively. **(B)** The landscape of electrostatic interaction energies. The location of the predicted orientation is marked by the red dot. **(C)** Left: predicted placement of the Cas9 complex [different viewpoint compared to the front view in **(A)**] highlighting the gluing role of guide RNA and the parallel orientation of the bound target DNA strand within the Cas9 protein relative to AFM substrate. For better visualization of the DNA located inside the protein, the Cas9 structure is shown in transparent. Middle: The scanning view perspective indicating the position of the two target-DNA gates together with the corresponding simulated AFM topography. Rotation of the scanning view orientation around the *z*-axis [compared to that shown in **(A)**], corresponding to a change in the viewpoint, is indicated. Right: HS-AFM image of the Cas9 complex with the DNA strand observed at locations very similar to predicted gates [adapted from Shibata et al. (2017)].

We first considered the Cas 9 endonuclease protein which binds guide RNA and cleaves duplex target DNA with a sequence complementary to the RNA guide, playing a key role in genetic engineering applications (CRISPR-Cas9 genome editing). Several PDB structures of Cas9 complexes are available. Figure 2A shows the atomistic structure of Cas9-RNA with a bound single-strand target DNA together with the computed molecular surface representation colored according to the calculated electrostatic potential. The presence of nucleic acid strands with the phosphate groups in nucleotides generates a negatively charged molecular surface. HS-AFM experiments to visualize structural dynamics of the Cas9-RNA-DNA complex (Shibata et al., 2017) were therefore performed on a modified surface known as APTES-mica which has a positive charge distribution (see Methods). Figure 4A shows the predicted molecular placement on the supporting surface predicted from our electrostatic interaction model. As can be seen from the bottom view perspective, the Cas9 complex binds to APTES-mica with the flat molecular surface that has the negatively charged guide RNA strand attached, which acts like a glue between Cas9 and APTES-mica (Figure 4C). The scanning view showing the molecular surface probed by the AFM tip is also provided (Figure 4A).

Looking closer at the predicted orientation of the Cas9 complex relative to the APTES-mica surface, an interesting observation can be made. The bound target DNA strand is located in a tunnel within the Cas9 structure and both the entry and exit paths are not blocked by contacts with APTES-mica. In fact, the orientation of both DNA gates is roughly parallel to the surface (Figure 4C). We then generated a simulated AFM image of the predicted orientation of the molecular structure in the scanning view perspective and compared it with a snapshot obtained from HS-AFM imaging the dynamics of Cas9 interactions with DNA (Shibata et al., 2017). As we find, in the experimental image the orientation of the DNA strand in the Cas9 complex correlates remarkably well with the position of the two DNA gates in the predicted molecular orientation. It should also be noted that the predicted molecular orientation of Cas9 relative to the AFM surface based on electrostatic modelling agrees remarkably well with our previous result (Amyot and Flechsig, 2020), where automatized rigid-body fitting of the Cas9 structure without the nucleic acids was employed to validate the domain arrangement seen in HS-AFM imaging.

Figure 4B shows the landscape of electrostatic interaction energies in the space of latitude and longitude angles ∅,θ which characterize the protein orientation in 3D space. The landscape shows a clear valley localized around the minima which corresponds to the predicted favorable placements of the Cas9-RNA-DNA structural template. This valley is confined by steep walls characterized by the gradient ∂V/∂ϕ which corresponds to the force magnitude of a perturbation that would be required to destabilize the placement on the protein on the APTES-mica surface. Hence, the presence of such a barrier would resist possible perturbations applied by the AFM tip in the horizontal scanning direction. However, the model simplifications underlying our predictions (see Discussion) do not allow to provide quantitative estimates that could be compared with those obtained from HS-AFM experiments.

Nonetheless, our findings based on electrostatics offer an explanation why under HS-AFM observations functional relative motions of target DNA and Cas9 can be reliably observed, and DNA cleavage could be captured at the single molecule level (Shibata et al., 2017).

### DNA-aptamer protein complex

Next, we considered a complex of a 70-nucleotide DNA aptamer and the CYP24 protein, which has been demonstrated to be relevant for antiproliferative activity in cancer cells and was previously observed under HS-AFM (Biyani et al., 2022). The 3D atomistic structure of the complex as predicted from molecular docking simulations and the computed molecular surface representation with charge coloring according to the electrostatic potential are shown in Figure 2B. Similar to the previous case of the Cas9-RNA-DNA complex, the presence of the DNA aptamer which due to the phosphate groups in nucleotides is negatively charged, does generally not allow stable AFM observations on the standard negatively charged bare mica surface. Therefore, the experiment was conducted with a mica surface modified by a positively charged lipid bilayer which self-assembled on its top [for details see Biyani et al. (2022)].

**FIGURE 5:**
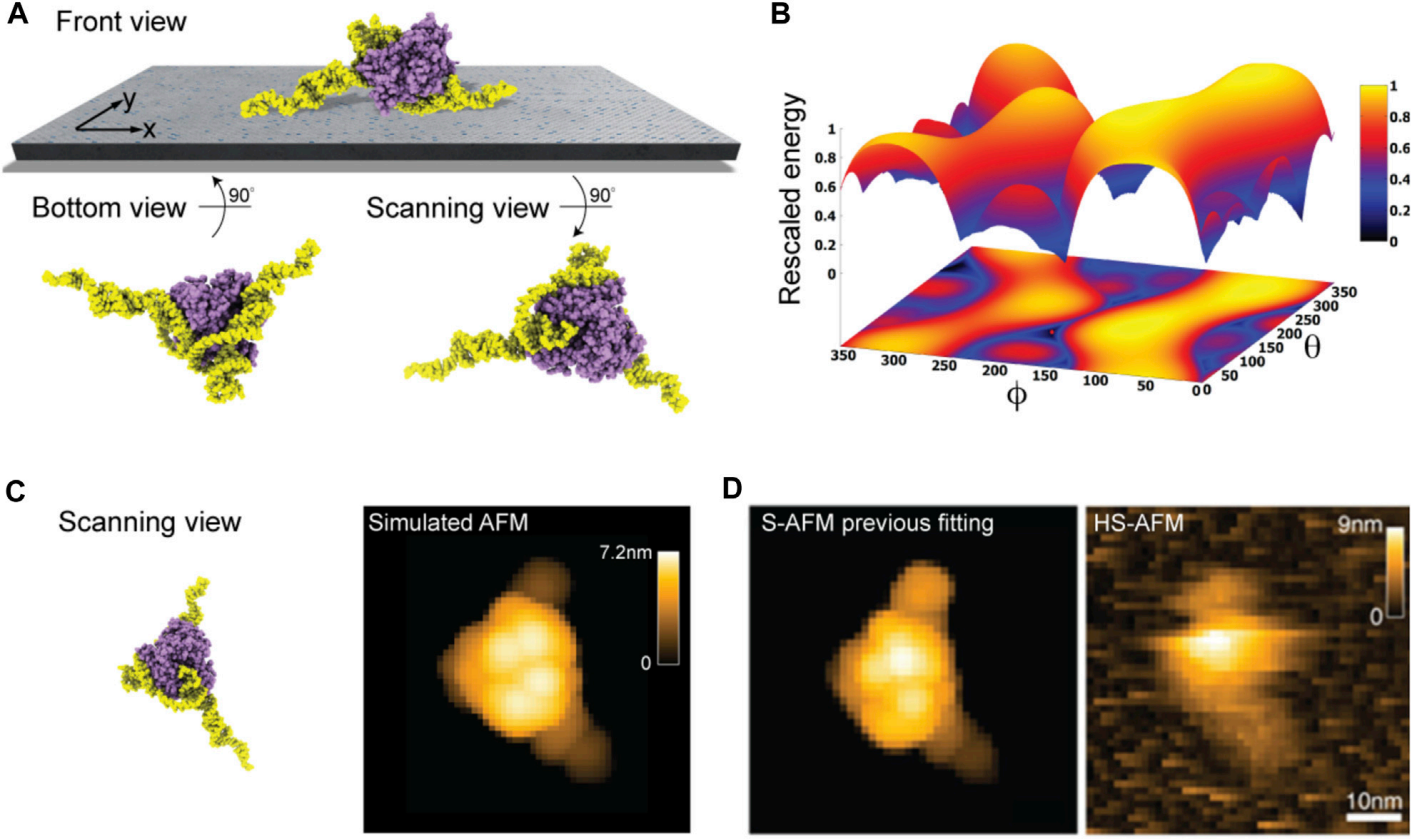
DNA-aptamer CYP24 protein complex. **(A)** Predicted orientation of the protein complex shown on the supporting surface in the front view. Additionally, the bottom view perspective displays the structure facing the surface and the scanning view shows the side probed by the AFM tip. **(B)** The landscape of electrostatic interaction energies. The location of the predicted orientation is marked by the red dot. **(C)** Scanning view of the predicted orientation [rotated around the *z*-axis compared to that shown in **(A)** to change the viewpoint] and the corresponding simulated AFM image. **(D)** Simulated AFM image of the molecular orientation (left), identified from previous fitting to a HS-AFM target image (right) based on exhaustive search [images adopted from Biyani et al. (2022)]. With permission, Copyright 2022 American Chemical Society).

Taking into the account the used lipid mixture (i.e., ∼ 90% DPPC and 10% DPTAP type) in our model of the lipid-bilayer substrate (see Methods) and electrostatic interactions with the aptamer-protein complex, we predict the favorable molecular placement. Figure 5A shows the top candidate found from scanning the space of possible rigid-body orientations relative to the surface and evaluating electrostatic interaction energies. As can be seen in the front view perspective, and particularly well from the bottom view, the predicted orientations are those in which the two longer DNA strands are placed on the surface in a flat arrangement with the CYP24 protein sitting on top. The scanning view showing the molecular surface probed by the AFM tip is also provided.

Figure 5B shows the landscape of electrostatic interaction energies between the aptamer-protein complex and the modified mica surface in the space of latitude and longitude angles ∅,θ . It shows a highly localized valley which is confined by steep and high walls and at its bottom has energy minima that correspond to the well-defined placement of the protein-DNA complex predicted from them. The existence of such a highly confined deep energy valley is clearly due to the presence of the DNA aptamer, dominating the electrostatic interactions by attraction forces with the positively charged lipid bilayer on mica. Any structural orientations deviating from the predicted highly stable conformation would be practically impossible, which is confirmed by single molecule HS-AFM observations of the CYP24-aptamer [see SI movie in Biyani et al. (2022)].

When comparing a simulated AFM image obtained in the scanning perspective of the predicted protein complex relative to the AFM substrate (Figure 5C) to our previous result from automatized fitting (Amyot and Flechsig, 2020) and a measured HS-AFM image (Figure 5D), excellent agreement is found.

### Monalysin pore-forming toxin

The third application is for the bacterial Monalysin protein which has been identified to form pores in cell membranes, thus contributing to the death of fruit flies (Opota et al., 2011). HS-AFM has previously revealed the structure of Monalysin in solution on a mica surface and on an effectively negative charged lipid membrane (Nonaka et al., 2020). For the application of our model, we have used the crystal structure of the pro-form Monalysin (PDB 4MJT), representing however the functionally inactive state (Monalysin active structures are missing). As a structural template of the active state we have used a nonameric structure with removed N-terminal segments, roughly taking into account their cleavage upon activation [see Leone et al. (2015) and Nonaka et al. (2020) for details].

Figure 2C shows the ring-shaped atomistic structure and the corresponding electrostatic surface representation. In this orientation the molecular surface exhibits a ring-shaped region with positive electrostatic potential. However, a significant area of the surface at the opposite protein side is also positively charged. Hence, which orientation can be expected to be preferential under the formation of the sample-substrate complex is unclear.

**FIGURE 6:**
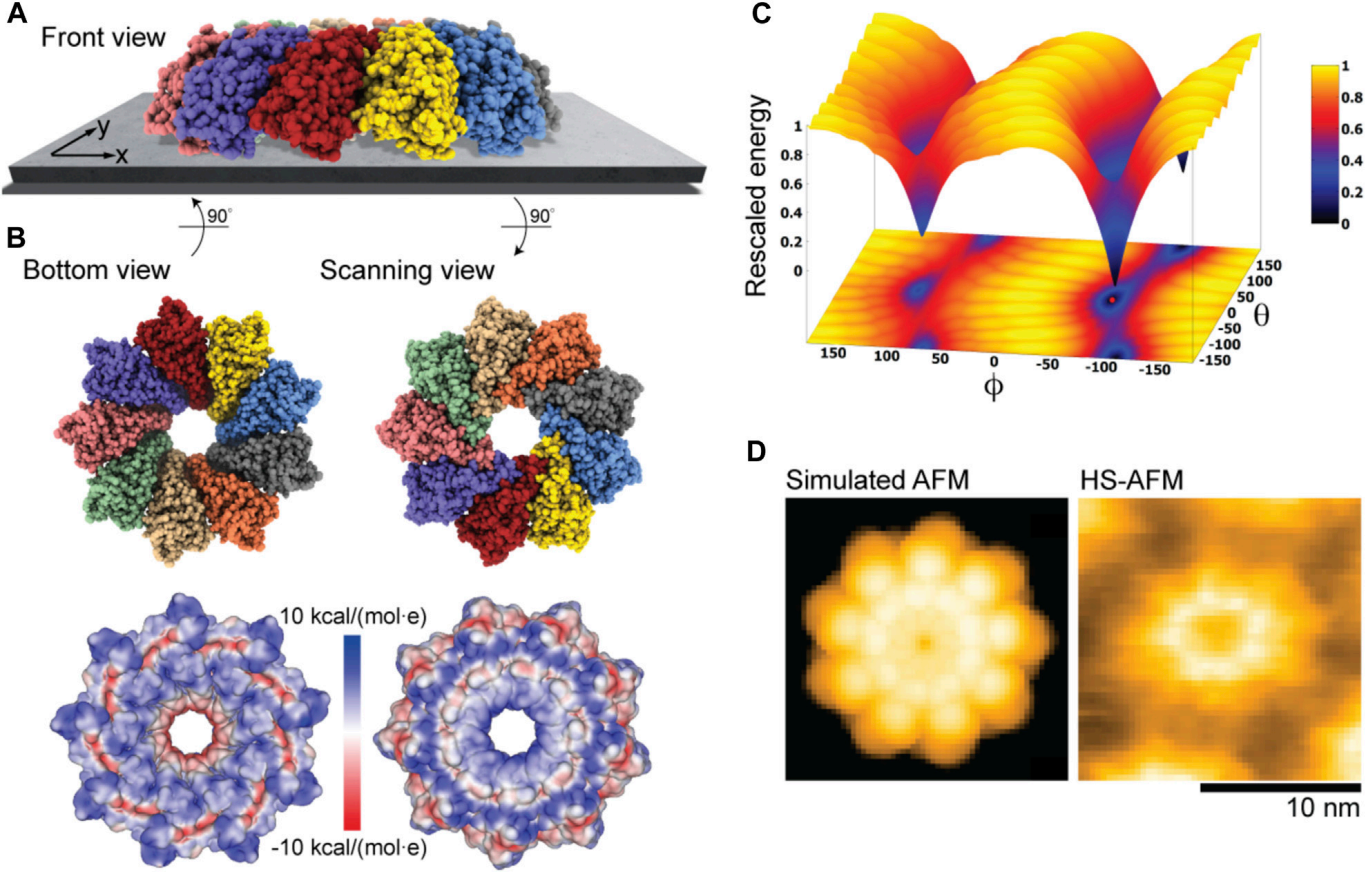
Monalysin pore-forming toxin. **(A)** Predicted dome-shape orientation of the Monalysin oligomer on the AFM substrate in the front view perspective. **(B)** Bottom view perspective displaying the atomic structure facing the surface and the corresponding molecular surface representation with colors indicating electrostatic potential values (left). Additionally, the scanning view perspective is provided (right). **(C)** The landscape of electrostatic interaction energies. The location of the predicted orientation is marked by the red dot. **(D)** Simulated AFM image of the scanning view perspective (left) and a HS-AFM image of Monalysin from previous experiments (Nonaka et al., 2020) (right).

Our electrostatic interaction model indeed predicts those two placements of Monalysin on mica to be the only stable states, as can be seen from the landscape of electrostatic interaction energies (Figure 6C). The favorable placement corresponds to that of the oligomer in the dome-shape shown in Figure 6A. The corresponding molecular structure in the bottom view perspective showing the interface towards the substrate is displayed in Figure 6B together with the surface representation colored according to the electrostatic potential. It can be concluded that the positively charged regions at the tip of monomers, whose arrangement resembles the jags of a crown, play a dominant role in the interactions with the AFM substrate and the sample placement. Images for the scanning view perspective are also shown in Figure 6B. Simulations considering the membrane model as a substrate with the lipid mixture according to the experimental conditions (i.e., ∼80% DOPC and 20% DOPS type) used in Nonaka et al. (2020) resulted in very similar predictions.

An important observation is that our predictions are consistent with the currently available models of membrane pore formation by the Monalysin protein (Leone et al., 2015; Nonaka et al., 2020), stating that binding to the membrane in the dome-shaped orientation is required to generate further conformational changes leading to the formation of nanopores. Furthermore, a simulated AFM image generated for the scanning view perspective of the predicted Monalysin orientation relative to the AFM substrate shows good agreement with the HS-AFM image obtained from previous experiments (Figure 6D). The differences in the size of simulated and measured topographies are attributed to the fact that the experiments visualize the active Monalysin as an octamer, whereas the used model structure is a nonamer and the conformational changes underlying a transition to the active form cannot be resolved.

### ClpB molecular chaperone

**FIGURE 7:**
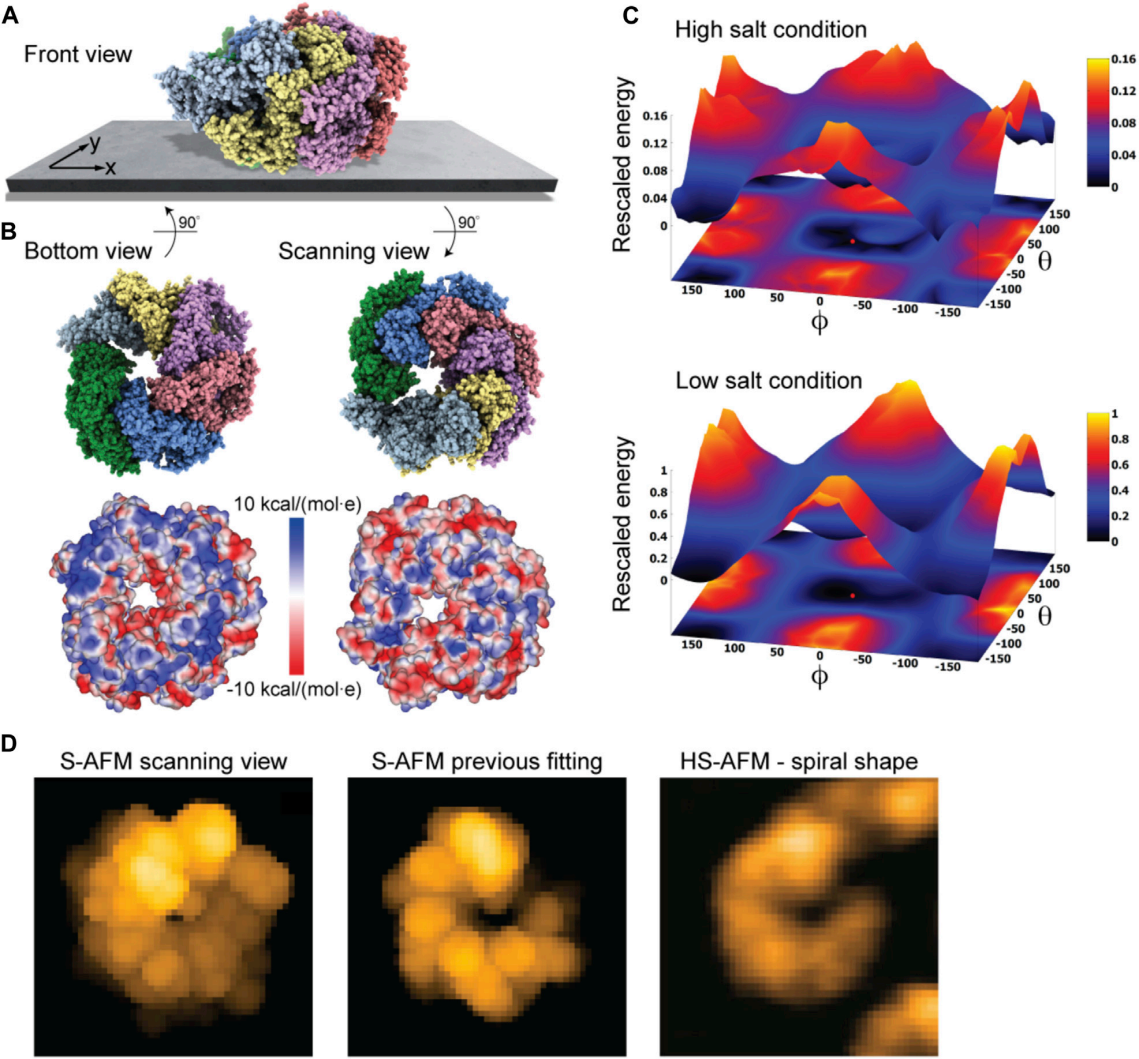
ClpB molecular chaperone. **(A)** Predicted orientation of the Hsp104 protein structure shown on the supporting surface in the front view. **(B)** Bottom view perspective displaying the atomic structure facing the surface and the corresponding molecular surface representation with colors indicating electrostatic potential values (left). Additionally, the scanning view perspective is provided (right). **(C)** The landscape of electrostatic interaction energies computed for high and low salt buffer conditions (top and bottom, respectively). The red dot marks the location of the predicted orientation. In both plots a common energy scale was used by rescaling. **(D)** Simulated AFM image of the predicted orientation in the scanning view perspective [in a viewpoint different from that in **(B)**, left]. Simulated AFM image of the molecular orientation (middle), identified from previous fitting to a HS-AFM target image [right, taken from Uchihashi et al. (2018b)] based on exhaustive search.

As a last application, we chose the ClpB chaperone which is an ATP-powered molecular machine involved, e.g., in disaggregation of proteins under heat stress conditions. Functional conformational dynamics of ClpB was previously investigated in HS-AFM experiments (Uchihashi et al., 2018b). The atomistic structure of the hexameric Hsp104 disaggregase (yeast homologue of bacterial ClpB) in the conformation with bound ATP analog is shown in Figure 2D. In the chosen orientation, the molecular surface representation reveals a ring-shaped region with predominantly positive electrostatic potential. It can therefore be expected that this protein side forms contacts with the mica surface. Our electrostatic interaction model employing a bare mica surface as used in ClpB HS-AFM observations indeed predicted orientations with similar contact surfaces. A single chosen predicted placement is shown in Figure 7A in the front view. The corresponding bottom view perspective displaying the protein side facing the mica surface together with its molecular surface representation colored according to the electrostatic potential is shown in Figure 7B. Also shown is the molecular structure in the opposite view corresponding to the scanning view perspective together with its surface representation (Figure 7B). As can clearly be seen, the ring-shaped region with predominantly positive electrostatic potential is guiding the placement of the hexameric chaperone on the negatively charged mica surface.

An interesting aspect is that for successful imaging of ClpB under HS-AFM the buffer composition was critical as stated in Uchihashi et al. (2018b): “The salt concentration was a key to successful imaging of TClpB because high salt concentrations such as 150 mM KCl weakened the affinity of molecules to mica substrate, resulting in fast diffusion of molecules and thus hampering imaging. Therefore, we used a lower concentration of KCl (20 mM) which enabled moderate binding of ClpB onto mica substrate.” While in the employed electrostatic model the buffer conditions can only be phenomenologically accounted for by the parameter for the ionic strength, our predictions can still provide an explanation of this situation. In Figure 7C we show the landscape of electrostatic interaction energies between the Hsp104 structural template and the mica surface for two cases, corresponding to high- and low-salt concentrations, respectively. Common to both landscapes is the presence of a valley isolating favorable protein placements on mica. However, the stark difference between them is that under low-salt conditions, electrostatic interaction energies, and therefore the barriers around the valley, are larger by one order of magnitude as compared to the high-salt case. This is because in the latter case, electrostatic interactions are screened over a much shorter Debye length (see Eq. 1). Hence, the interpretation is that a buffer condition with low-salt concentration significantly stabilizes the formation of electrostatically favorable orientations of ClpB on mica and therefore allows reliable imaging under HS-AFM scanning. Notably, the landscape of electrostatic interaction energies resembles that obtained for the toy *Janus sphere* (Figure 1D), reflecting the stability of the sample-substrate complex in the obtained orientation, whereas the placement in the opposite upside-down protein arrangement represents the most unstable arrangement.

The simulated AFM image of the predicted orientation resembles the spiral shape topography seen in HS-AFM imaging (Figure 7D), which arises from the domain protrusions in the hexameric arrangement. Interestingly, our previous result of automatized fitting the PDB structural template into the same HS-AFM image predicted the hexamer structure to be in the opposite upside-down orientation. There, fitting was based on exhaustive sampling of possible molecular orientations without an underlying physical model, aiming to identify the orientation whose simulated AFM image best matched to the target HS-AFM image. In fact, the thus obtained simulated AFM image matches much better to the HS-AFM image compared to the one obtained from our electrostatic model predictions (Figure 7D). A well-known drawback in the interpretation of results is that simulated topographies (like the measured AFM topographies) have a limited spatial resolution. Especially for symmetrically shaped proteins this may lead to ambiguities. While the atomistic structure on opposite sides of the ClpB ring is clearly distinct, the corresponding simulated AFM images resulting from a convolution of the tip shape with the molecular structure can show similar looking spiral shapes.

Predictions based on our electrostatic model should in principle be prioritized over the sampling method without any physical interactions. However, a drawback of the modeling is that in the absence of structural ClpB data used in HS-AFM experiments (*T. thermophilus* ClpB) the yeast homologue Hsp104 structure was used. Therefore, the expected differences in the sequence may also result in a different surface electrostatic potential compared to ClpB and predictions of the sample-substrate complex will change.

A so far overlooked issue is that HS-AFM observations were performed under a high protein concentration imaging assembly of ClpB rather than single molecules. Therefore, additional inter-molecular interactions may influence the placement on the mica substrate and imaging stability.

## Discussion

We address a simple question relevant in all biomolecular scanning probe experiments: can the sample placement on the supporting substrate be predicted?—with the answer obviously being, of course. Our approach based on electrostatic interactions allows such predictions considering available structural data prior to an actual experiment. We demonstrated its validity in applications to HS-AFM imaging to not only confirm resolution-limited imaging results using atomistic-level information, but also to offer an explanation about the stability of observations. Buffer conditions are considered in the model by phenomenologically including, e.g., the salt concentration via the ionic strength in the Debye-Hückel form of interactions, and the pH value affecting the biomolecular surface electrostatic potential.

Providing models of AFM substrates and considering physical interactions with the biomolecular sample, our approach complements previously developed computational methods to infer 3D atomistic biomolecular conformations from resolution-limited experimental AFM imaging (Dasgupta et al., 2020; Niina et al., 2020; Dasgupta et al., 2021; Niina et al., 2021), which neglected modelling of sample-substrate interactions.

Apparently, the presented model implies gross simplifications. The rigid-body approximation of the biomolecular sample neglects any possible internal conformational motions. In that sense, the presented approach builds on our previous work on rigid-body sampling to infer atomistic structure from AFM images by automatized fitting (Amyot et al., 2022).

Nonetheless, conformational motions can play a role in interactions with the AFM substrate. In flexible regions near the molecular surface, for example, structural changes will likely occur when electrostatic interactions with the substrate set in. The formulated models of AFM substrates are also a simplification of the complex molecular arrangement. e.g., it was to our surprise that although the APTES-mica substrate is widely used in HS-AFM experiments, the mechanism underlying adsorption of APTES molecules on the mica surface seems to be largely unexplored and we could construct only a rough model. On the other side, as we have found for the two cases involving mica (Monalysin and ClpB), within the approximations of our model the predictions of biomolecular placement do not depend on the details of the substrate model and the characteristics of the computed electrostatic interaction landscapes are qualitatively similar. However, the studied examples represent rather large samples with distinct charge patterns on their molecular surfaces. For smaller biomolecular samples and those with finer charge patterns, our atomistic model of the mica substrate surface considering the detailed representation of the charge geometry shall allow for a refinement of predictions compared to the coarse-grained lattice model.

For the electrostatic interactions between the sample and the AFM substrate, long-range interactions are taken into account as the dominant contribution underlying sample placement, whereas, consistent with the approximate nature of our approach, short-range interactions are neglected.

As we demonstrated in this work, efficient predictions which agree remarkably well with experimental observations can be obtained despite the plethora of approximations. However, the provided example applications can obviously not be generalized and limitations as to what extent static structural data can be employed are expected, especially in applications of highly flexible proteins. Furthermore, since our coarse-grained modelling does not allow to infer a realistic magnitude of electrostatic interaction energies, the interpretation about imaging stability is only qualitative and does not allow quantitative comparison to the forces exerted by an AFM tip.

While the emphasis of our approach is to allow for computationally efficient predictions, higher-resolution models which consider molecular dynamics of the sample, refined modelling of various AFM substrates, and a more detailed description of sample-substrate interactions (also those beyond electrostatics) shall be constructed in the future to provide improvements of predictions and widen the margin of applications.

The developed methods are implemented in our BioAFMviewer package freely available at www.bioafmviewer.com, allowing for convenient applications within a well-established user-friendly interactive software interface. We are inviting the Bio-AFM community to use the new tool and are anticipating constructive feedback.

## Data Availability

The authors state that the data supporting the findings of this study are available within the article.
